# Himadri Sarkar: Premature demise of a genius

**DOI:** 10.4103/0970-1591.56172

**Published:** 2009

**Authors:** Arunava Choudhury

**Affiliations:** Professor of Urology, Vivekananda Institute of Medical Sciences, Rama Krishna Mission Seva Pratishthan, 99 Sarat Bose Road, Kolkata 700026, India. E-mail: arunava.choudhury@gmail.com

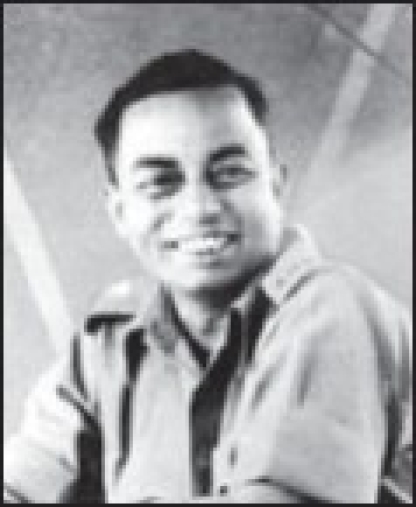



**Himadri Sarkar**

In 1960, when Urology had been established in the USA for barely over a decade and half the 22 medical colleges in the U.K. did not have a department of genitourinary surgery, a few intrepid surgeons of India joined together to form the Urology Section of the ASI (Association of Surgeons of India). Among these pioneers was a man from the east of the nation – diminutive in frame, but lofty in stature – Himadri Kumar Sarkar. For him, it was not new being a founder member, since a couple of years before that, the urologists of Calcutta under the Presidentship of Kumar Kanti Ghosh (one of the early presidents of the USI), had banded together to form the Urology Association of Calcutta, and its founder secretary was Dr. Himadri Sarkar.

Dr Himadri Sarkar was born on 20 October, 1920 in the small hill town of Kurseong, near Darjeeling, where his father was engaged in legal practice. He spent his childhood in Kurseong and had his schooling in the northern suburbs of the city of Calcutta. He studied Intermediate in Science in the St. Paul's College in Calcutta. He rode with distinction through these formative years to enter the Calcutta Medical College in 1940. This institution was one of the two earliest medical colleges of Asia (established in 1835), and had very high standards. He was an outstanding student of his generation and again carried before him most of the prizes, gold medals and scholarships that were available to him during the study period of six years. He later qualified as a doctor in 1946.

He had the good fortune to train as house surgeon to the legendary Col. Anderson, the famous British surgeon of the Medical College Hospitals, Calcutta, and mentor to many an aspirant surgeon of the time. He proceeded to UK where he worked in Bradford, under the redoubtable Harry Hamilton Stewart, the pioneer of genitourinary surgery in northern England, and it was he who kindled in Himadri Sarkar the interest in urology. By 1949, he had obtained his FRCS from both the English and the Edinburgh colleges and returned with pride to an India that had gained its independence by then.

On his return, he accepted a position as a whole time surgeon in the large hospital of the Calcutta Port Commissioners, which was then the largest port of the country. He gained enormous experience in different branches of surgery in this hospital, and his early publications reflect both the variety and the quality of his work: be it gastric surgery, intestinal surgery, limb trauma or spinal surgery. He received wide acclaim for his thesis on the clinico-pathological study of cervical spinal cord injuries: an interest developed as he would have to manage, in the Port hospital, unfortunate ship porters with paralysis following neck injuries while carrying a load on the head. He would travel from morgue to morgue in the city at odd hours of the night attending post mortems of patients dying from such injuries and performed painstaking dissection of cadaver specimens. This was research of high order and was an object lesson in how much could be achieved with limited facilities. Deservedly he won for himself the coveted ‘Sankaran Memorial Prize’ of the Association of Surgeons of India for his efforts in 1954.

He wished to enter the field of academic medicine, and much as he loved the Port Hospital, he would now have to leave it. He joined the Post Graduate Medical Institute in Kolkata in 1957 as Assistant Professor of Surgery. This was an honorary position. Here he subsequently developed his abiding interest in the urological sciences. At that time, in Calcutta – after Dr. Kumar Kanti Ghosh, he was the only second surgeon who showed an interest in pure urology rather than a mix of urology and general surgery; the director of surgery at the time, the distinguished surgeon Dr. Ajit Kumar Basu allowed him to run his unit as a Urology unit from 1961.

He had led a varied existence over a short span of life. In 1960, his patriotic sense forced him to join the Territorial Army, where he reached the rank of Major. This was initially limited to weekend exercises, but later this interrupted his academic activities for a while, as he took active part in the 1962 Sino-Indian conflict in the North-East Frontier Agency, and worked in the army hospitals at Kolkata and Jabalpur. He fell in love with the discipline of work that existed in the army, and the lifestyle of an army officer. He collected books on war surgery to refine his skills and totally absorbed himself in his new role. An avid photographer, he maintained on his Rolleiflex exciting photographic records of wartime NEFA, the Naga locals and the famous Stilwel road.

Urology beckoned, and he left the territorial army in 1963 and rejoined the IPGMER, Kolkata, and pressed forwards with his dream of establishing a formal urology unit. Government approval was finally obtained in 1964. This was the time that he reached the pinnacle of his career. With his wonted and infectious enthusiasm, he developed the specialty of urology quickly to remarkable heights. He was a master surgeon, direct in his approach, practical in his ways, deft of fingers, sure of knowledge and confident in the outcome of whatever he dealt with. His peers remember that it was a pleasure to see him tackle difficult and intricate problems in urology. He was a hard working man, always willing to learn from others and trying to improve himself. He invested in a tape recorder in the early 1960s, a novelty at the time, so that he could rehearse and re-rehearse his speeches and perfect his oratory. Always unassuming, he shunned undue publicity. Meticulous in keeping records of his patients, his clinical presentations were always enriched with both true and authentic clinical facts and experience. He had the uncanny knack of being able to discard the unimportant aspects of a truly difficult problem and quickly go into the hard core that really mattered.

A Kolff dialysis unit was installed as early as 1963 at the post graduate institute – it was the one of the very few such machines in India at the time; regular dialysis were carried out and he was in the planning process to start kidney transplantation. Though the M.Ch course was still decades away, due to his popularity and dynamism, every year he would get three or four students under his wings, for working on their thesis for M.S. examination of the Calcutta University. Within five years there were 14 theses guided by him., He also started research programs on his own on the subjects of his interest, like genitourinary tuberculosis, pathophysiology of the bladder neck obstruction, renal artery stenosis, renal tumours etc. Out of some 35 papers, his article on GU Tuberculosis was a seminal one for the period (Genitourinary tuberculosis in male; Calcutta Medical Journal, December 1960). As one of the postgraduate teachers of the nation, he was invited to be examiner in Madras and Vellore, which had started their postgraduate training programs. He was keen to upgrade the unit into a department and visited Dr. H.S. Bhat in 1965 to obtain details of the CMC Vellore organization. Finally, in 1968, the government accepted the proposal of upgradation of the unit to a department.

In the year 1965, the urology section of the ASI decided to hold its third annual conference in Calcutta and quite wisely chose Himadri Sarkar to be the Organizing secretary and his mentor Kumar Kanti Ghosh was its president. The venue expectedly was IPGMER. His tremendous enthusiasm and dynamism produced an excellent conference with a very large number of delegates coming from different urological centers of the country, which the old-timers still remember fondly.

He built up, over the years, an immense private practice, attracting patients from all over India and neighboring countries. His skills in prostate surgery, partial nephrectomy and reconstruction for tuberculosis were legendary. He maintained high standards of ethics in his practice. He would preserve detailed records of his consultations and surgical procedures, all neatly filed and bound for posterity. At his home he acquired a large library of medical books dating from the 19th century as well as very contemporary texts in urology.

For close to a decade, Himadri Sarkar was the nucleus of development and hopes and aspirations of a whole generation of Calcutta surgeons aspiring to be urologists, who looked upto him for guidance. He was a very straightforward person brooking no nonsense, and would not mind calling a spade ‘a spade’. He was also a strict disciplinarian but truly had an iron hand and a velvet glove. He would occasionally, in the same breath, admonish a junior for some shortcoming and praise him for good work. His students fondly say that they forgave him for his vicious scoldings for he had a ‘heart of gold’.

Most of his planned research projects were yet to reach their culmination, when came a bolt from the blue. In early 1970, he developed jaundice that was diagnosed to be a case of carcinoma of the head of the pancreas. At operation only a biliary-enteric bypass was possible, and this allowed him a few months of active life. He knew of the diagnosis and its consequences. He had always been a man in hurry; but in these last few months of his life, he plunged headlong into the hospital work in one hand and private practice on the other hand – concerned about providing for his very young family. The end came on 6th October 1970, a few days before his 50th birthday. This was not just a personal loss for his family and friends, but also a severe setback to the development and establishment of urology in the East. It was more than two decades after his death, when the first M.Ch. course in eastern India was finally started in the department developed by him.

Himadri Sarkar was married to Shefali, an artist, and an illustrator in many of his surgical papers. They had three children. He would spend his weekends at his farmhouse with his family, far away from practice pressures; here he would indulge in his love for agriculture and outdoor pursuits. His early death was a blow for his then young family, and his wife, a lady of extraordinary fortitude, guided the children through their lives. She died in 2006. Devoted to the memory of her husband she had carefully preserved his career documents and his library. If alive, he would have been proud to see that his three children are all very successful in their chosen careers, and that his only son became an urologist; he would also have been delighted to see that he has six grandchildren to whom his life has been inspirational. Four of his five grandsons have chosen the medical profession, with two of them already training to be surgeons.

It does come across that Himadri Sarkar was a very special person with greatness built into him. Coupled with this was an enormous zest for life and work that left its mark on his peers and students. Almost forty years after his death, he is still remembered with respect in the city both in the medical professional world and in society: a true legend.

The Urological Society of India – in its wisdom, instituted an oration – the first and the most prestigious one of the society, Himadri Sarkar Memorial Oration, which is delivered by the immediate past president of the society every year during the annual conference.

